# Effects of urban coarse particles inhalation on oxidative and inflammatory parameters in the mouse lung and colon

**DOI:** 10.1186/s12989-017-0227-z

**Published:** 2017-11-22

**Authors:** Cécile Vignal, Muriel Pichavant, Laurent Y. Alleman, Madjid Djouina, Florian Dingreville, Esperanza Perdrix, Christophe Waxin, Adil Ouali Alami, Corinne Gower-Rousseau, Pierre Desreumaux, Mathilde Body-Malapel

**Affiliations:** 1 Inserm, CHU Lille, U995-LIRIC-Lille Inflammation Research International Center, Univ. Lille, F-59000 Lille, France; 2 Inserm U1019, CNRS UMR 8204, Institut Pasteur de Lille– CIIL – Center for Infection and Immunity of Lille, Univ. Lille, F-59000 Lille, France; 3 SAGE - Département Sciences de l’Atmosphère et Génie de l’Environnement, IMT Lille Douai, Univ. Lille, 59000 Lille, France

**Keywords:** Particulate matter, Coarse PM, Oxidative stress, Inflammation, Gut-lung axis, *N*-acetyl-L-cysteine

## Abstract

**Background:**

Air pollution is a recognized aggravating factor for pulmonary diseases and has notably deleterious effects on asthma, bronchitis and pneumonia. Recent studies suggest that air pollution may also cause adverse effects in the gastrointestinal tract. Accumulating experimental evidence shows that immune responses in the pulmonary and intestinal mucosae are closely interrelated, and that gut-lung crosstalk controls pathophysiological processes such as responses to cigarette smoke and influenza virus infection. Our first aim was to collect urban coarse particulate matter (PM) and to characterize them for elemental content, gastric bioaccessibility, and oxidative potential; our second aim was to determine the short-term effects of urban coarse PM inhalation on pulmonary and colonic mucosae in mice, and to test the hypothesis that the well-known antioxidant *N*-acetyl-L-cysteine (NAC) reverses the effects of PM inhalation.

**Results:**

The collected PM had classical features of urban particles and possessed oxidative potential partly attributable to their metal fraction. Bioaccessibility study confirmed the high solubility of some metals at the gastric level. Male mice were exposed to urban coarse PM in a ventilated inhalation chamber for 15 days at a concentration relevant to episodic elevation peak of air pollution. Coarse PM inhalation induced systemic oxidative stress, recruited immune cells to the lung, and increased cytokine levels in the lung and colon. Concomitant oral administration of NAC reversed all the observed effects relative to the inhalation of coarse PM.

**Conclusions:**

Coarse PM-induced low-grade inflammation in the lung and colon is mediated by oxidative stress and deserves more investigation as potentiating factor for inflammatory diseases.

**Electronic supplementary material:**

The online version of this article (10.1186/s12989-017-0227-z) contains supplementary material, which is available to authorized users.

## Background

Episodic increases in ambient air contaminant levels have been demonstrated to modulate the pathogenesis of an increasing number of chronic diseases, from asthma to cancer and stroke [1]. Exposure to ambient air pollution, especially to particulate matter (PM), is a major risk factor for pulmonary diseases such as asthma, chronic bronchitis, and pneumonia [2]. Numerous epidemiological studies indicate that long- and short-term exposure to coarse PM is associated with adverse health effects on the human respiratory system [3].

Although research on airborne pollutants has focused mostly on cardiovascular and respiratory effects, emerging epidemiological and experimental evidence suggests that air pollutants can also cause adverse effects on the gastrointestinal tract. Recent epidemiological studies have reported that exposure to air pollution may be associated with various gastrointestinal diseases including inflammatory bowel diseases [4, 5], appendicitis [6], irritable bowel syndrome [7], and enteric infections in children [8]. Notably, a correlation has been reported between ambient air pollution and hospitalizations for inflammatory bowel diseases in Wisconsin [9].

To date, only one study has assessed the effects of PM inhalation on the gastrointestinal tract in an animal model [10]. Li et al. reported that in Ldlr^−/−^ mice, inhalation of ultrafine PM led to shortened villus length, which was accompanied by prominent macrophage and neutrophil infiltration into the ileum. Ultrafine PM exposure also increased the concentrations of intestinal free oxidative fatty acids and lysophosphatidic acids. This study was the first to report that inhaled particles can trigger a deleterious effect at the intestinal level, which justifies the value of exploring this topic further.

Investigations of the mechanisms responsible for air pollution exposure-induced toxicity are challenging because of the complexity of air pollutants. Reactive gases such as ozone, nitrogen oxides, carbon monoxide, sulfur dioxide, volatile organic compounds and PM of varying size are part of the air pollutant mixture. The physicochemical characteristics of this complex matrix of gases and PM are also highly variable depending on the generation mode and sources (e.g., point industrial sources, automotive combustion, natural processes such as wildfires and volcanic eruptions, and atmospheric conditions) [11]. Air particles are known sinks for various organics molecules and a number of inorganic chemicals including physiologically active transition metals.

Our study focused on coarse PM, which has an aerodynamic diameter between 2.5 and 10 μm. It therefore includes an extra-thoracic particulate fraction (particles from 5 to 10 μm in size) and a thoracic particulate fraction (particles from 2.5 to 5 μm in size) [12]. The particulate fraction deposited into the extra-thoracic region becomes trapped in the nasal cavity, mouth, and pharynx. The vast majority of particules deposited in the extrathoracic region are removed via a combination of nose-blowing, sneezing, and mucociliary transport to the gastrointestinal tract [13]. Ingestion is therefore the dominant exposure pathway to particles deposited in the extra-thoracic region. Meanwhile, the thoracic particulate fraction deposits in the tracheobronchial region. This region consists of trachea, bronchi and terminal bronchioles. These particles, trapped in the mucus produced by the bronchial epithelial cells are typically cleared by mucociliary transport into the throat, and then expectored or swallowed [14, 15]. Moreover, for both fractions, soluble particles can be absorbed directly via the airway epithelium and cleared into the blood or lymphatic system [13, 16]. Therefore, coarse PM appears to be a relevant PM fraction to study, because it comes into direct contact with the digestive tract and has several input pathways allowing it to affect both the pulmonary and intestinal mucosae.

The first objective of this study was to characterize metal content of urban coarse PM collected in the French city of Douai, hereafter called coarse PM^D^ (cPM^D^) and to assess its gastric metal bioaccessibility. Because oxidative stress is a major mechanism of PM toxicity, the oxidative potential of cPM^D^ was quantified. The second aim was to assess the effects of inhalation of cPM^D^ at a concentration relevant to episodic elevation peak of air pollution in mice. The third aim was to evaluate the effect of the administration of a well-known antioxidant, the N-acetyl-L-cysteine (NAC), on the effects of cPM^D^ inhalation in mice.

## Methods

### Sample collection

Aerosol samples were collected with a High-Vol (35 m^3^/h) six- stages (10.2, 4.2, 2.1, 1.4, 0.73, 0.41 μm) cascade impactor (Tisch Environmental Inc.). Particles were collected during the warm season from July 13 to October 9, 2013, in Douai, a small city in a densely urbanized region in the north of France, located about 100 km from the English Channel coast (Additional file 1: Figure S1). Daily meteorological data during the sampling period were retrieved from the nearest weather station of the French meteorological service *Météo-France*, located 20 km north of the sampling site at the airport of Lille-Lesquin. PM was collected onto each impactor plate covered with adhesive Teflon stripes, which allowed the particles to be easily swiped with a Teflon tip, and then transferred directly into acid-cleaned sterile tubes, weighed, homogenized, and kept at 4 °C until later sampling for chemical and biological assays. The particle fraction size of the cPM^D^ used in this study was between 2.1 and 10.2 μm.

### Total metal concentration

Particulate trace element mineralization and analysis were performed in triplicate, following a previously published method [17]. About 3 mg of cPM^D^ was acid digested in a microwave oven (Milestone ETHOS) at 220 °C. Digests were diluted to 50 mL with ultrapure water and analyzed by inductively coupled plasma mass spectrometry (ICP-MS) (NeXion 300XX, PerkinElmer) for trace elements (As, Ba, Bi, Cd, Ce, Co, Cr, Cs, Cu, Hg, La, Li, Mn, Mo, Ni, Pb, Rb, Sb, Sc, Se, Sn, Sr, Th, Ti, Tl, U, V, Zn) and major elements (Al, Ca, Fe, K, Mg, Na). An internal mixed standard (69Ga, 103Rh) was added (1 μg/L) to all analyzed solutions to correct the drift of the ICP-MS signal. Reagent blanks, quality controls and standard reference materials (NIST 1648a and ERM CZ-120) were also analyzed repeatedly to validate the entire analytical procedure, as previously described [18]. The total metal concentration is expressed in micrograms of metal per gram of cPM^D^.

### Metals bioaccessibility

Gastric bioaccessible fractions of cPM^D^ were characterized after in vitro extraction using a synthetic gastric juice (SGJ), according to the previously described procedures [19]. The gastric extractions were performed using three aliquots of 1–5 mg of cPM^D^ by agitating the PM suspensions for 2 h at 37 °C in accordance with the estimated physiological residence time. The separation of the particles from the synthetic fluid was performed by centrifugation for 10 min at 14,600 rpm at 6 °C. The supernatant was analyzed after a HNO_3_ digestion on a hot plate, evaporation to dryness, and dilution to 10 mL (1% HNO_3_). The results are expressed in micrograms of solubilized metal in the supernatant per gram of cPM^D^. To check the consistency of the measured concentrations, the residual fraction was acid digested in a microwave oven at 220 °C according to the method of Alleman et al. [17]. Elemental analyses were performed in triplicate. Trace and major elements were validated through repeated analysis of reagent blanks, quality controls and standard reference materials (NIST 1648a and 2584). The bioaccessible fractions are presented as the ratio (expressed as a percentage) of metal concentration measured in the gastric leaching solutions to the total metal concentration.

### Oxidative potential of cPM^D^ measured in ascorbic acid (AA) depletion assays

Oxidative potential is defined as a measure of the capacity of PM to oxidize target molecules, here AA, by generating reactive oxygen species in an acellular assay [20]. The AA depletion assay was performed under physiological conditions: at 37 °C and pH 7.4 in potassium Phosphate-buffered saline (PBS) at 10^−2^ mol/L, containing 30% KH_2_PO_4_ and 70% K_2_HPO_4_ on a molar basis, that had been pretreated with Chelex resin to remove all potential metallic contaminants. Ten milligrams of cPM^D^ was solubilized in 300 mL of PBS 10^−2^ mol/L in an ultrasonic bath for 30 min and then agitated at 37 °C for 24 h. The cPM^D^ suspension was prefiltered through a 0.45 μm syringe filter and divided into three cPM^D^ extracts of 100 mL each. Next, 0.5 mL of AA at 4.10^−2^ mol/L in PBS was added to each cPM^D^ extract and the sample was mixed. The absorbance was measured at 265 nm for different times up to 1 h for both the cPM^D^ extract solution test and the blank samples without cPM^D^. The molar concentration of AA was then calculated from the absorbance at 265 nm using a preestablished calibration curve. A faster AA depletion rate in the presence of cPM^D^ extract compared with the blank indicates that cPM^D^ promotes the oxidation of AA. A similar assay using 10 mg of cPM^D^ in 300 mL of PBS 10^−2^ mol/L was performed in the presence of 87.5 mg of EDTA, a transition metal chelating agent, before introducing the AA reactant to examine the effect of metal redox activity on AA depletion (Zielinski et al., 1999). All experiments were performed in triplicate. Oxidative potential is expressed as the maximum AA depletion rate in micromoles per liter per minute (i.e., the depletion rate calculated during the first hour) calculated after subtracting the blank for a solid-to-liquid ratio of 10 mg-to-300 mL.

### Animals

The animal treatment protocol was approved by the regional bioethics committee (committee no.75; authorization no.CEEA2016072517274040) and all of the animals received human care in accordance with the Guide for the Care and the Use of Laboratory Animals (National Research Council (US) Committee 2011).

**Scheme 1:**
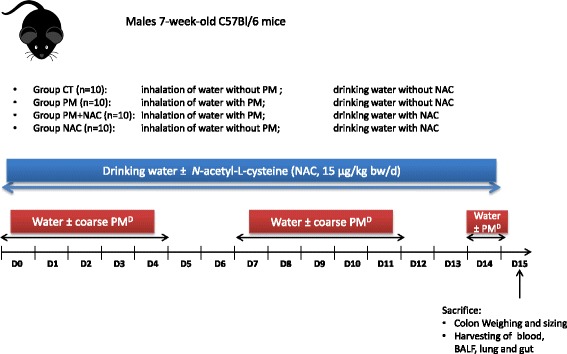
Experimental design of the mouse model

Male C57BL/6 mice (aged 7 weeks) were purchased from Janvier Labs and housed under standard conventional conditions. The room relative humidity was 55% and the temperature was 21 °C. Mice were randomly divided into the different exposure groups (*n* = 10/group), as described in Scheme 1 and Fig. 4a.

### Inhalation exposures

For inhalation experiments, mice were placed 4 h/day, 5 days/week for 2 weeks and for one additional day in a ventilated whole-body inhalation chamber that allowed free movement (InExpose, Scireq®) [21]. Nebulization was achieved using an Aeroneb Lab™ ultrasonic nebulizer directly connected to a 5 l–chamber and controlled through the flexiWare software v.6 according to the following parameters: bias flow of 2 l/min, nebulization rate of 0.083 ml/min, which were measured and controlled throughout the experiment. The Aeroneb Lab™ ultrasonic nebulizer produced droplets with a volume median diameter of 11 μm characterized by laser diffraction using the Spraytec system from Malvern Instruments. Mice were exposed to a solution of 40 μg cPM^D^/ml or to the soluble or insoluble fractions of cPM^D^. The dose concentration of the aerosol achieved with these conditions was 1.66 μg/L. Control mice were exposed to sterile water under the same conditions. cPM^D^ suspension was fractionated into soluble and insoluble fractions by centrifugation for 5 min at 13000 g as previously described [22, 23]. Soluble and insoluble fractions were diluted in the same volume of sterile water as the total fraction.

### N-acetyl-L-cysteine (NAC) administration

Mice were administrated NAC (Sigma-Aldrich) in drinking water (15 μg/kg/day), from the beginning of the PM exposure until the day of sacrifice as described in Scheme 1.

### Biological samples

Mice were euthanized the morning following the final exposure day. The colon was dissected and measured. Then, the colon was emptied by pushing the stool outwards using a dissecting forceps, and weighed. Bronchoalveolar lavage fluid (BALF) and samples of the lungs, colon, and blood were collected and kept on ice for FACS analysis or immediately frozen at −80 °C.

### Flow cytometry

Cells harvested from BALF or extracted from lung tissue were washed in PBS and incubated with antibodies (BD, Franklin Lakes, NJ, USA) for 30 min in PBS and then washed twice and suspended in PBS with 2% fetal calf serum. The antibodies used were: fluorescein isothiocyanate (FITC)-conjugated anti-I-A[b]; phycoerythrin (PE)-conjugated anti-F4/80; peridinin chlorophyll protein complex (PerCP)/Cy5-conjugated anti-CD103; PE/Cy7-conjugated anti-CD11c; allophycocyanin (APC)-conjugated anti-CCR2; Alexa 700-conjugated anti-CD86; APC-H7-conjugated anti-Ly6G; V450-conjugated anti-CD11b; V500-conjugated anti-CD45; BV605-conjugated anti-Ly6C; FITC-conjugated anti-CD5; PE-conjugated anti-CD1d tetramer; PerCP/Cy5-conjugated anti-NK1.1; APC-conjugated anti-CD25; Alexa 700-conjugated anti-CD69; APC-H7-conjugated anti-CD4; V450-conjugated anti-T-cell receptor-β; V500-conjugated anti-CD8, and BV605-conjugated CD45. Cells were analyzed on an LSR Fortessa cell analyzer (BD). The generated data were analyzed using FlowJo 8.7 (TreeStar, Stanford, CA, USA).

### Gene expression in tissues

Total mRNA from lung and colon tissues was extracted using a Nucleospin RNA II kit (Macherey Nagel). Reverse transcription was performed using a High Capacity cDNA Archive Kit and quantitative polymerase chain reaction (PCR) with SYBR Green (Life Technologies). The primer sequences were designed using Primer Express 3 (Life Technologies) and are available upon request. Melting curve analyses were performed for each sample and gene to confirm the specificity of the amplification. Because the exposure to PM did not cause any significant alterations in *Polr2a* mRNA expression, the relative expression of each gene of interest was normalized to the relative expression of this gene. The quantification of the target gene expression was based on the comparative cycle threshold (Ct) value. The fold changes in the target genes were analyzed by the 2^−ΔΔCt^ method [24].

### Serum malondialdehyde (MDA) analysis

Serum samples (50 μL) were incubated with acetic acid and SDS at 95 °C for 1 h, followed by centrifugation at 800 g for 10 min. Supernatants were transferred to a 96-well plate and the absorbance was measured at λ_ex_ = 532 and λ_em_ = 553 nm. 1,1,3,3-Tetramethoxypropane (Sigma-Aldrich) was used as a standard. Protein concentration in samples was determined using a DC™ protein assay (Bio-Rad Laboratories). Serum MDA concentration was corrected by the sample protein concentration and is expressed as nanomoles per milliliter of serum.

### Myeloperoxidase activity assay

Neutrophil influx into colon was analyzed by measuring the enzymatic activity of myeloperoxidase (MPO). Mice colons were homogenized in 0.5% hexadecyltrimethylammonium bromide (Sigma-Aldrich) in 50 mM PBS, freeze-thawed three times, and centrifuged. MPO was analyzed in the clear supernatant by adding 1 mg/mL of dianisidine dihydrochloride (Sigma-Aldrich) and 5.10^−4^% hydrogen peroxide (H_2_O_2_), and the change in optical density was measured at 450 nm. Human neutrophil MPO (Sigma-Aldrich) was used as a standard. One unit of MPO activity was defined as the amount that degraded 1 μmol of peroxide per min at 25 °C. Readings from tissue samples were normalized to total protein content as detected in the DC™ protein assay (Bio-Rad).

### Statistical analysis

Results are expressed as mean ± SEM. The statistical significance of differences between experimental groups was calculated using the Mann–Whitney U test (GraphPad, San Diego, CA).

## Results

### Characteristics of cPM^D^

The meteorological conditions during the sampling period were typical for summertime under the oceanic climate of the northwestern European coast. The daily averages were a temperature of 17.7 °C (Additional file 1: Figure S2A), 71% relative humidity (Additional file 1: Figure S2B), 1017 hPa atmospheric pressure and cumulated rain of 172 mm over the whole period (Additional file 1: Figure S2C). These relatively high pressures and sparse rainfall conditions were observed throughout the period, and a continuing decrease in temperature and increase in relative humidity were recorded from summer to mid-autumn. The average wind speed was 3.3 m/s (Additional file 1: Figure S2D) and winds came mostly (39%) from the west sector (marine air masses from the English Channel and Atlantic Ocean) and secondarily (28%) from the north sector (more continental air masses toward the regional *Scarpe-Escaut* natural park and the south of Belgium (Additional file 1: Figure S2E).

**Fig. 1:**
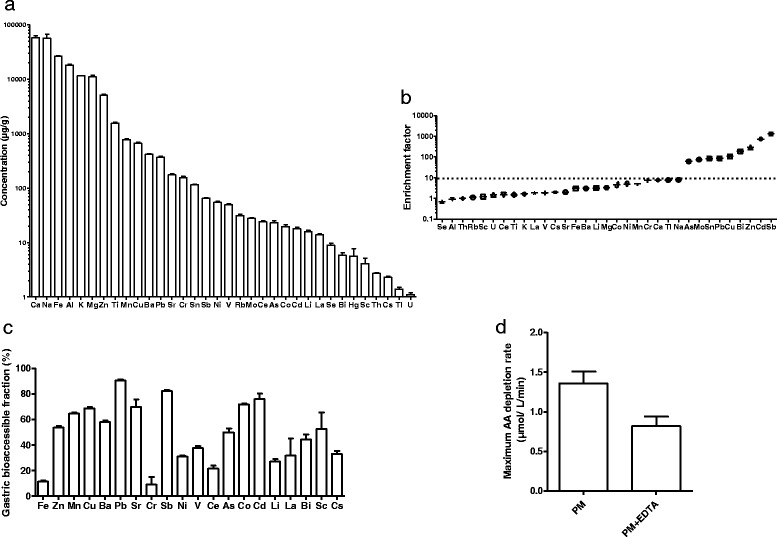
Coarse PM^D^ characterization. **a** Mean concentrations of trace and major elements (μg/g). **b** Enrichment factors relative to Thorium. **c** Gastric bioaccessibility in SGJ (%). **d** Measurement of oxidative potential of cPM^D^ in the AA depletion assay with or without EDTA, and for a solid-to-liquid ratio of 10 mg/300 mL, expressed as the maximum rate of AA depletion (μmol/L/min)

The concentrations of elements in cPM^D^ varied from 1 to 57,312 μg/g in the following order: U < Tl < Cs < Th < Sc < Hg < Bi < Se < La < Li < Cd < Co < As < Ce < Mo < Rb < V < Ni < Sb < Sn < Cr < Sr < Pb < Ba < Cu < Mn < Ti < Zn for trace elements and Mg < K < Al < Fe < Na < Ca for major elements (Fig. 1a). Enrichment factors relative to the upper continental crust with Th as the reference element [25] were applied to assess the anthropogenic influence on cPM^D^ content (Fig. 1b). Enrichment factors >10 were observed for As, Mo, Sn, Pb, Cu, Bi, Zn, Cd, and Sb. All of these elements are commonly associated with traffic nonexhaust emissions (i.e., brakes and tire wear), particularly Cu, which is predominant in coarse PM in Europe [26], and Sb, which showed the highest enrichment factor, and confirmed the urban typology of the sampling site. Cd, Zn, Bi, and Pb are known tracers of nonferrous smelting [27–29] and their enrichment may indicate the influence of a nearby Zn smelter located 3 km north of our sampling site. To characterize cPM^D^ better, we sought to assess their features in relation to other urban coarse PM. For this purpose, the elemental concentrations in cPM^D^ were compared with those measured in other European cities: an urban site in Helsinki, Finland [30] and a traffic site in Budapest, Hungary [31] (Additional file 1: Figure S3). Globally, the elemental concentrations were very similar, except for the highest levels of Cd, Zn and Bi in Douai, in accordance with a significant influence of the nearby Zn smelter.

### Gastric bioaccessibility of cPM^D^

Simulation of solubilization pathways was performed using the SGF to approximate gastric conditions (Fig. 1c). Metals showing the higher gastric bioaccessibility were Pb > Sb > Cd > Co > Sr, with values ranging from 90% for Pb to 70% for Sr. The lowest gastric bioaccessibility were observed for Cr (9%) and Fe (11%).

### Oxidative potential of cPM^D^

The oxidative potential of cPM^D^ was assessed using an AA depletion assay (Fig. 1d); AA is an antioxidant naturally present in the human body. The rates of AA depletion measured showed the effective oxidative potential of cPM^D^. To examine the effects of metal redox activity on AA depletion, the same experiment was performed in the presence of EDTA, a transition metal chelating agent. In the presence of EDTA, AA depletion was significantly lower (− 42%) although not totally depleted, which indicates a role of metals in the oxidative potential of cPM^D^.

### Effects of cPM^D^ inhalation on oxidative stress and inflammation in mice

**Fig. 2:**
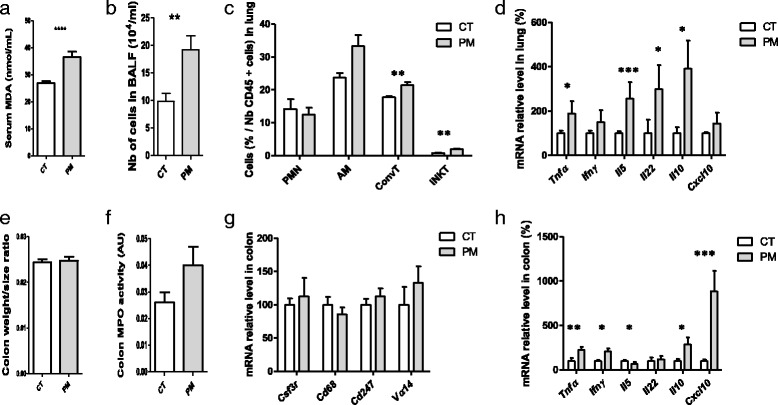
Effects of cPM^D^ inhalation on oxidative stress and inflammation in mice. Mice that inhaled cPM^D^ were compared with control mice that inhaled water (CT). **a** Serum MDA concentration. **b** BALF cellularity. **c** Counts of polymorphonuclear neutrophils (PMN), alveolar macrophages (AM), conventional T cells (ConvT) and iNKT cells in the lung determined by flow cytometry. **d** Quantitative PCR (qPCR) analysis of cytokine and chemokine mRNA levels in the lung. **e** Colon weight/size ratio. **f** Colon MPO activity. **g** qPCR analysis of mRNA levels of PMN, macrophage, T cell and iNKT cell markers in the colon. **h** qPCR analysis of cytokine and chemokine mRNA levels in the colon. Data are presented as mean ± SEM. *p < 0.05, **p < 0.01, ***p < 0.001, Mann–Whitney U test

We then exposed mice to this cPM^D^ by inhalation in a whole body inhalation chamber. By this physiological way of exposure, as in real-life, coarse PM is expected to deposit primarily in the upper respiratory tract, and then to be transported from the conducting airways to the gastrointestinal tract by mucociliary clearance [12, 32]*.* We first aimed to assess the effects of an environmentally relevant episodic increase in ambient PM exposure on pulmonary and intestinal mucosal tissues. Following 14 days of exposure to inhaled cPM^D^, serum MDA concentration was significantly higher in the PM mice compared with CT mice that inhaled only water. This finding reflects the appearance of systemic oxidative stress (Fig. 2a). More cells were obtained in BALF from PM mice compared with CT mice (Fig. 2b). The numbers of neutrophils and alveolar macrophages in the lung did not differ significantly between PM and CT mice (Fig. 2c). By contrast, more conventional T cells and invariant natural killer (iNKT) cells were observed in the lung of PM mice, and these increased cell numbers were associated with higher *Tnfα*, *Il5*, *Il22,* and *Il10* mRNA levels (Fig. 2d). At the intestinal level, the colon weight size ratio did not differ between PM and CT mice (Fig. 2e). A trend toward an increase in MPO activity was observed in the colons of PM mice (Fig. 2f). The gene expression levels of markers of neutrophils (*Csf3r*), macrophages (*Cd68*), conventional T cells (*Cd247*), and iNKT cells (*Vα14*) did not differ between PM and CT mice (Fig. 2g). By contrast, significantly greater expression of *Tnfα*, *Ifnγ, Il10* and *Cxcl10*, and lower expression of *Il5* were found in the colons of PM mice compared with CT mice (Fig. 2h). Together, these results suggest that cPM^D^ inhalation led to low-grade inflammation in both the lungs and gut.

### Effects of NAC administration on cPM^D^ -induced deleterious effects

**Fig. 3:**
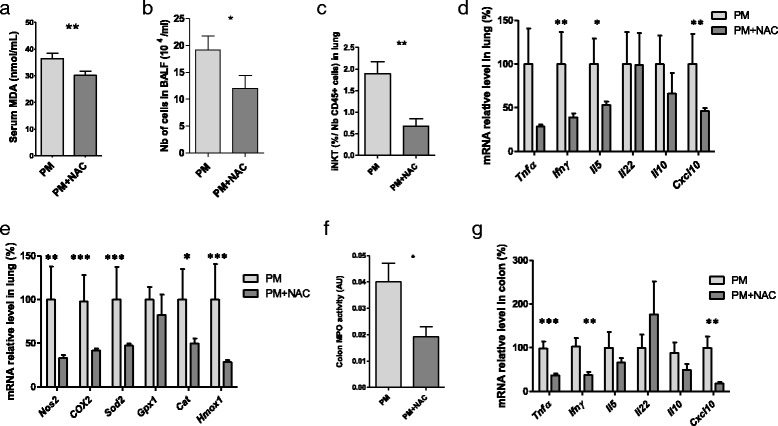
Effects of NAC administration on cPM^D^-induced damage. All mice inhaled PM. Mice that received NAC (15 μg/kg/day for 14 days) in drinking water were compared with CT mice that did not receive NAC in drinking water. **a** Serum MDA concentration. **b** BALF cellularity. **c** iNKT cell count in the lung measured by flow cytometry. **d** qPCR analysis of cytokine and chemokine mRNA levels in the lung. **e** qPCR analysis of mRNA levels of oxidative stress markers in the lung. **f** Colon MPO activity. **g** qPCR analysis of cytokine and chemokine mRNA levels in the colon. Data are presented as mean ± SEM. *p < 0.05, **p < 0.01, ***p < 0.001, Mann–Whitney U test

Oral administration of NAC has been shown to protect against oxidative stress in the lung [33, 34]. We evaluated the consequences of NAC administration on the previously observed PM-induced effects. NAC was added to drinking water of mice that inhaled PM (PM + NAC mice). A group of mice that inhaled PM and did not receive NAC in drinking water was also included (PM mice). Serum MDA concentration was lower in PM + NAC mice than in PM mice (Fig. 3a). Fewer cells were found in BALF from PM + NAC mice than in that from PM mice (Fig. 3b). In the lungs, less iNKT recruitment (Fig. 3c) and lower *Ifnγ*, *Il5* and *Cxcl10* expression levels were observed (Fig. 3d). The mRNA levels of several oxidative stress markers such as *Nos2*, *COX2*, *Sod2*, *Cat*, and *Hmox1* were markedly lower in the lungs of PM + NAC mice compared with those from PM mice (Fig. 3e). The colons of PM + NAC mice showed lower MPO activity (Fig. 3f) and reduced *Tnfα*, *Ifnγ* and *Cxcl10* mRNA expression compared with those from PM mice (Fig. 3g). Taken together, these data show that NAC administration reversed the deleterious effects induced by PM inhalation. NAC did not alter the basal condition because NAC treatment did not modify inflammatory and oxidative stress parameters in serum, lung, or colon of CT mice that inhaled water (Additional file 1: Figure S4).

### Involvement of water-soluble and insoluble fractions on cPM^D^-induced low-grade inflammation

**Fig. 4:**
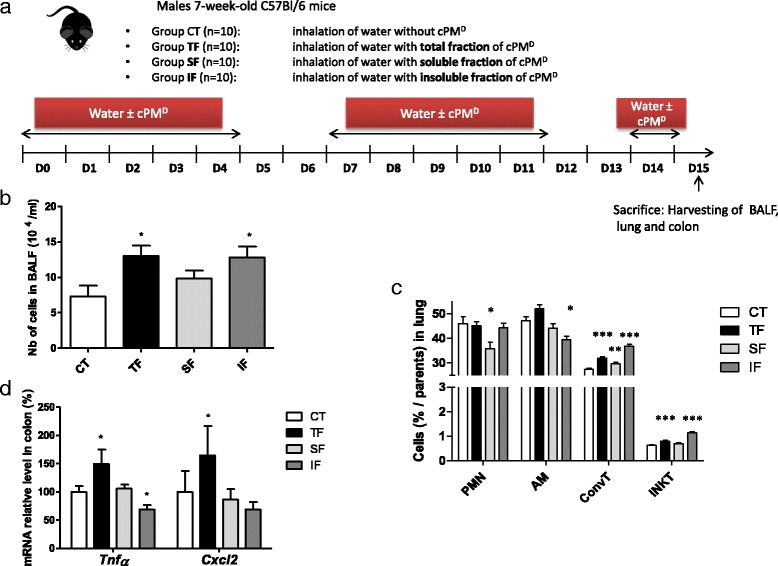
Involvement of water-soluble and insoluble fractions on cPM^D^-induced low-grade inflammation. **a** Mice experimental protocol. **b** BALF cellularity. **c** Counts of polymorphonuclear neutrophils (PMN), alveolar macrophages (AM), conventional T cells (ConvT) and iNKT cells in the lung determined by flow cytometry. **d** Quantitative PCR analysis of *Tnfα* and *Cxcl2* mRNA levels in the colon. Data are presented as mean ± SEM. *p < 0.05, **p < 0.01, ***p < 0.001, Mann–Whitney U test

To assess if the solubilization of PM in water during nebulization is involved in the observed low-grade inflammation, an experiment was performed including in addition to the 2 previously described groups [mice exposed to water (CT group), and mice exposed to cPM^D^ (total fraction, FT group)], a group exposed to the soluble fraction of cPM^D^ (Soluble Fraction, SF group) and a group exposed to the insoluble fraction of cPM^D^ (Insoluble Fraction, IF group) (Fig. 4a). The increased number of cells in BALF in TF-exposed mice compared to CT mice was found also in IF-exposed mice, and not in SF-exposed mice (Fig. 4b). Similarly, the enhancement of iNKT cells in lungs of TF-exposed mice compared to CT mice was also found in IF-exposed mice, and not in SF-exposed mice (Fig. 4c). In the colon, an unexpected decrease of *Tnfα* levels was quantified in IF-exposed mice compared to CT mice (Fig. 4d). Most interestingly, no increase of *Tnfα* and *Cxcl2* mRNA levels was detected in the colons of SF-exposed mice, while the transcription of both genes was increased in TF-exposed mice. Taken together, these results show that the water-soluble fraction of cPM^D^ is not sufficient to induce the low-grade inflammation associated to cPM^D^ inhalation.

## Discussion

The cPM^D^ used in this study comprised urban particles collected over 4 months of the warm season in northern France, and ranged in size from 2.1 to 10,2 μm. Fe, Cu, and Zn were among the most concentrated metals quantified in cPM^D^, and their high concentrations are of concern because of their pro-oxidative potential [35]. The most enriched element was Sb, which is consistent with the main traffic-related origin of cPM^D^. Another metal of concern is Pb, which is highly toxic and is enriched by 86 times in cPM^D^ [36, 37]. Both Sb and Pb concentrations were similar to those reported for other urban areas [30, 38, 39]. The strong enrichment for Cd and As in cPM^D^ compared with the upper crust is likely to contribute to their negative health impact. Cd exposure is known to induce pulmonary damage such as emphysema and lung cancer [40, 41] and to cause intestinal inflammation in mice [42]. Similarly, As exposure has been repeatedly associated with lung carcinogenesis [43] and has been shown to perturb the gut microbiome in mice [44].

Furthermore, Pb, Sr, Sb, Co and Cd presented both high concentration in cPM^D^ and high gastric bioaccessibility. A substantial proportion of these elements is therefore likely to be found in the bloodstream during air pollution peak in urban areas. The diffusion of these metals in the bloodstream could lead to both directly cytotoxic effects on circulatory monocytes [45], but also to indirectly aggravating effects on inflammation in peripheral tissues: notably, lead and cadmium levels in PM have been found negatively associated with miR-146a expression in blood leukocytes RNA from foundry workers [46]. Mir-146a is involved in limiting inflammatory responses triggered through the innate immune system [47]. Moreover, miR-146a-mediated NOD2-SHH signaling regulates gut inflammation in murine model of inflammatory bowel diseases [48].

The main objective of the current study was to assess the effects of cPM^D^ inhalation at both the pulmonary and colonic levels. Our in vivo study was conducted in conditions as close as possible to real life, and aimed to determine the effects of a realistic inhalation of coarse PM. Exposure in an inhalation chamber was preferred to oral or intratracheal administration. For their aerosolization, the cPM^D^ were solubilized in sterile water; the impact of solubilization on the observed effects seems negligible, since the mice that inhaled the water-soluble fraction of the cPM^D^ did not exhibit pulmonary and colon low-grade inflammation. The dosage of PM nebulized in the inhalation chamber is close to that breathed during pollution peaks by inhabitants of megacities strongly affected by particulate pollution. In urban areas, the mean daily concentration of PM of ≤10 μm in diameter (PM_10_) ranges from <10 μg/m^3^ to >200 μg/m^3^ [49]. In 2002, the US Environmental Protection Agency reported a range of maximal city PM concentrations of 26–534 μg/m^3^ [50]. Extreme hourly concentrations of PM_10_ reaching 800 μg/m^3^ have been measured at a traffic site in London [51]. PM_10_ pollution peaks at >250 μg/m^3^ were measured in megacities in India, Pakistan and China in 2010 [52, 53]. Therefore, the dose used here is fairly representative of high pollution episodes in the most affected megacities worldwide.

The studies performed until now that assess the effects of coarse PM at the pulmonary level were performed using intratracheal instillation or oropharyngeal aspiration [54–57]; these two modes of coarse PM administration partially or totally excluded the physiological exposure of the mouth, nose, larynx and pharynx. They revealed in most of the cases pulmonary inflammation, which the players were variable regarding the type of PM, the timing and the dose of exposure. Some consistencies can however be found with our study assessing the effects of coarse PM following inhalation. Tumor necrosis factor (TNFα) produced by activated alveolar macrophages and by epithelial cells is a master cytokine of the inflammation induced by PM in the lung [58, 59]. As for TNFα, increased levels of interleukin 5 (IL-5), IL-10, and IL-22 are in agreement with previous studies. Overexpression of both IL-5 and IL-10 in the lung has been shown following early life exposure to combustion-derived PM [58]. IL-22 upregulation may be linked to activation of the aryl hydrocarbon receptor (AHR), as reported by previous work showing the ability of urban dust PM to induce Th17 polarization and IL-22 production in an AHR-dependent manner [60]. The most pronounced effects on the recruitment of immune cells involved in pulmonary inflammation induced by cPM^D^ seemed to involve iNKT cells. Our report is the first to show that this immune population is associated with PM exposure, although its key role has been demonstrated in the pulmonary response to ozone [61] and cigarette smoke [62].

Few studies have assessed the effects of PM inhalation at the intestinal level. As expected because of the low dose of cPM^D^ used, we did not find evidence of colitis clinical manifestation in the PM-exposed mice, as reflected by the lack of effect on the colon weight size ratio. Accordingly, some major intestinal immune populations, namely neutrophils, macrophages, lymphocytes and iNKT cells, do not seem to be significantly affected by PM inhalation. However, the increase in colon MPO activity, which almost reached significance, as well as the strong increase in the colonic expression of *Tnfα*, *Ifnγ* and *Cxcl10* argue in favor of a low-grade inflammation generated in colon following PM inhalation. The large production of *Il10* may be indicative of a regulatory response, although the decrease in *Il5* level remains unexplained.

It is the first time that the effects of coarse PM administration on mice are studied by natural ventilation in an inhalation chamber. Nevertheless, when a high dose of coarse PM was administrated to mice by gavage during 14 days, an increase of pro-inflammatory cytokines (*Il12a*, *Il17,* and *Il2*) has been described [63]. Moreover, increases in colon TNFα and IFNγ protein levels have also been reported following 10 and 14 weeks of oral coarse PM intoxication in mice starting from the neonatal period [64]. Taken as a whole, the experimental protocols used to explore the effects of coarse PM on the gastrointestinal tract consistently describe low-grade intestinal inflammation, but are too diverse to reveal the common immune cells or molecular pathways implicated in the colonic effects of PM. Notably, it remains to be determined whether the observed effects on the gastrointestinal tract are mediated through a topical effect of PM, which comes into direct contact with intestinal epithelium, or through a systemic mechanism.

The triggering of low-grade intestinal inflammation could contribute in part to many health issues. Low-grade intestinal inflammation is a feature of irritable bowel syndrome [65]. It could also exacerbate Inflammatory Bowel Diseases [66], and promote colon carcinogenesis [67]. Moreover, a huge body of evidence indicates that low-grade intestinal inflammation participates in whole-body metabolism, and therefore to the metabolic syndrome that embraces cardiovascular diseases, type 2 diabetes, and non-alcoholic liver disease [68, 69]. It has also been speculated that intestinal low-grade inflammation associated with dysbiosis may play a pathophysiological role in human brain diseases, including autism spectrum disorder, anxiety, depression, and chronic pain [66, 70]. Intestinal low-grade inflammation has also been associated with chronic obstructive pulmonary disease [71]. Because of its critical role in health, any disturbance of intestinal immune homeostasis should be considered as a serious health threat.

A prominent finding of our study is that oxidative stress was a key mechanism for PM-induced deleterious effects. The AA depletion assay showed the oxidative potential of cPM^D^ and the involvement of metal content in this property. Consistently, PM metal chelation prevents the systemic inflammatory response induced in mice by repeated PM intratracheal instillations [72]. Quinones [73] and polycyclic aromatic hydrocarbons [74] present in PM are likely to also contribute to the oxidative potential of cPM^D^. The increase in serum MDA concentrations that we observed in cPM^D^-exposed mice reflects systemic oxidative stress, which is a well-known effect of PM in rats [75] and in humans [76]. NAC has been used for several years as an antioxidant agent [77–79]. In our study, NAC administration led to a normalization of pulmonary and systemic phenotypes. Our results lead us to hypothesize a similar mechanism to that demonstrated in chronic obstructive pulmonary disease induced in mice by cigarette smoke exposure [34]: that the protective effects of NAC are mediated through reduced accumulation and activation of iNKT cells. Most noticeably, NAC administration led to obvious protective effects in the colon, such as downregulation of MPO activity and complete reversal of the phenotype observed in PM-exposed mice compared with control mice, i.e., extinction of *Tnfα*, *Ifnγ*, and *Il10* upregulation. Because the concentration of NAC shown to induce significant improvement in colitis following oral administration (150 mg/kg body weight (bw)/day [80] or 160 mg/kg bw/day [81]) is much higher than that used in our study (15 μg/kg bw/day), these effects are not likely to be attributed to a topical effect of NAC.

NAC has been also described as a metal-chelating agent, able to increase urine gold excretion in rheumatoid arthritis patients treated with gold sodium thiomalate [82]. Moreover, intraperitoneal administration of NAC was very effective in increasing the urinary excretion of chromium and borate, but not lead, in rats intoxicated with potassium dichromate, boric acid and lead tetracetate respectively [83]. On the other hand, when NAC is administrated orally, its anti-oxidative properties are not systematically associated with chelating effects. By instance, in lead-exposed mice, oral administration of NAC (5.5 mmol/kg) reduced several indices of oxidative stress in both brain and liver samples, but not tissue lead levels [84]. Similarly, NAC treatment of arsenic-exposed rats (1 mmoml/kg) did not reduce significantly blood and liver arsenic levels, but reversed the elevation of brain MDA levels observed in untreated animals exposed to arsenic [85]. Especially, if the effects observed are mediated by the bioaccessible metal fraction of PM, it can be hypothesized that the attenuating effects of NAC are partly due to its metal chelating properties, but it is unlikely that this is the major mode of action of NAC.

Taken together, our results suggest that PM inhalation induces a succession of oxidative and inflammatory reactions that involve immune populations in the blood and in the pulmonary and gastrointestinal mucosae. Our work did not allow us to determine how these populations communicate but highlight an involvement of the gut-lung axis. This concept is strengthened by several lines of evidence showing the pathophysiological relevance of the immune crosstalk between the gut and lung. For example, house dust mite aeroallergens induce inflammation in the respiratory mucosa and reduce the gut epithelial barrier integrity [86]. In allergic airway disease, gut microbiota metabolism of dietary fibers influences the severity of allergic inflammation [87]. The importance of the gut microbiota has also been reported during respiratory influenza virus infection [88] and pneumococcal pneumonia [89]. The effects of cigarette smoke on the small intestine and colon, as evidenced by epithelial barrier defects, inflammatory cell recruitment, and microbial shifts, have been well described [62, 90, 91]. Therefore, our data demonstrating the deleterious effects of inhaled PM on both the pulmonary and colonic mucosae constitute additional evidence for the gut-lung axis, which deserves further investigation.

## Conclusions

In healthy mice, inhalation of urban coarse PM which presented with significant oxidative potential induced a low-grade inflammation that affected both the pulmonary and colonic mucosae. The development of this low-grade inflammation was at least partly driven by an oxidative stress mechanism, as evidenced by its reversal by concomitant administration of the antioxidative compound NAC. Our results provide further demonstration of a gut-lung axis, and highlight the importance of understanding the mechanisms of crosstalk between the pulmonary and colonic mucosae. Together with previous experimental and epidemiological observations, our study strongly suggests that coarse PM inhalation may trigger or accelerate the development of both pulmonary and gastrointestinal inflammatory diseases, particularly in genetically susceptible individuals.

## Additional file

Additional file 1:
**Figure S1.** Location of the sampling site. **Figure S2**. Meteorological conditions during the sampling period. **A** Temperature. **B** Relative humidity. **C** Rain. **D** Wind speed. **E** Wind direction and frequency. **Figure S3**. Comparison of trace element concentrations in cPM^D^ collected at Douai and coarse PM collected in Helsinki, Finland [30] and Budapest, Hungary [31]. **Figure S4**. Effects of NAC in the basal condition. All mice inhaled sterile water. Mice that received NAC (15 μg/kg/day for 14 days) in drinking water were compared with CT mice that did not receive NAC in drinking water. **A** Serum MDA levels. **B** iNKT cell count measured by flow cytometry. **C** Quantitative PCR (qPCR) analysis of cytokine and chemokine mRNA levels in the lung. **D** qPCR analysis of mRNA levels of oxidative stress markers in the lung. **E** MPO activity in the colon. **F** qPCR analysis of cytokine and chemokine mRNA levels in the colon. Data are presented as mean ± SEM. **p* < 0.05, Mann-Whitney U test. (DOCX 1064 kb)

## Abbreviations

AA: Ascorbic acid
AHR: Aryl hydrocarbon receptor
AM: Alveolar macrophage
APC: Allophycocyanin
BALF: Bronchoalveolar lavage fluid
Cat: Catalase
Cd: Cluster of differentiation
ConvT: Conventional T cell
COX2: Cytochrome c oxidase subunit II
cPM^D^: Coarse particulate matter from Douai
Csf3r: Colony stimulating factor 3 receptor
CT: Control
Cxcl10: C-X-C motif chemokine 10
Cxcl2: C-X-C motif chemokine 2
FITC: Fluorescein isothiocyanate
Gpx1: Glutathione peroxidase 1
Hmox1: heme oxygenase 1
ICP-MS: Inductively coupled plasma mass spectrometry
Ifnγ: Interferon gamma
Il: Interleukin
iNKT: Invariant Natural Killer T cell
MDA: Malondialdehyde
MPO: Myeloperoxidase
NAC: N-acetyl-L-cysteine
Nos2: Nitric Oxide Synthase 2
PE: Phycoerythrin
PerCP: Peridinin chlorophyll protein complex
PM: Particulate matter
PMN: Polymorphonuclear neutrophil
SGJ: Synthetic gastric juice
Sod2: Superoxide dismutase 2
Tnfα: Tumor necrosis factor alpha
Vα14: V alpha 14
