# Supplementary material for: Effects of urban coarse particles inhalation on oxidative and inflammatory parameters in the mouse lung and colon
Source: Part Fibre Toxicol. 2017 Nov 22;14:46. doi: 10.1186/s12989-017-0227-z (PMC5700563; doi:10.1186/s12989-017-0227-z)
Supplement: Additional file 1: — Figure S1. Location of the sampling site. Figure S2. Meteorological conditions during the sampling period. A Temperature. B Relative humidity. C Rain. D Wind speed. E Wind direction and frequency. Figure S3. Comparison of trace element concentrations in cPMD collected at Douai and coarse PM collected in Helsinki, Finland [30] and Budapest, Hungary [31]. Figure S4. Effects of NAC in the basal condition. All mice inhaled sterile water. Mice that received NAC (15 μg/kg/day for 14 days) in drinking water were compared with CT mice that did not receive NAC in drinking water. A Serum MDA levels. B iNKT cell count measured by flow cytometry. C Quantitative PCR (qPCR) analysis of cytokine and chemokine mRNA levels in the lung. D qPCR analysis of mRNA levels of oxidative stress markers in the lung. E MPO activity in the colon. F qPCR analysis of cytokine and chemokine mRNA levels in the colon. Data are presented as mean ± SEM. *p < 0.05, Mann-Whitney U test. (DOCX 1064 kb) [file 12989_2017_227_MOESM1_ESM.docx]

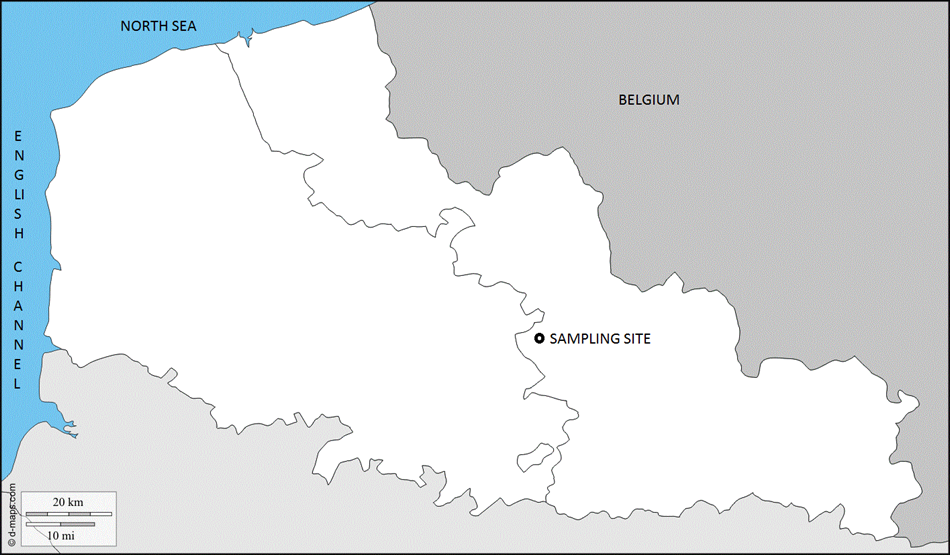


**Figure S1**. **Location of the sampling site.**


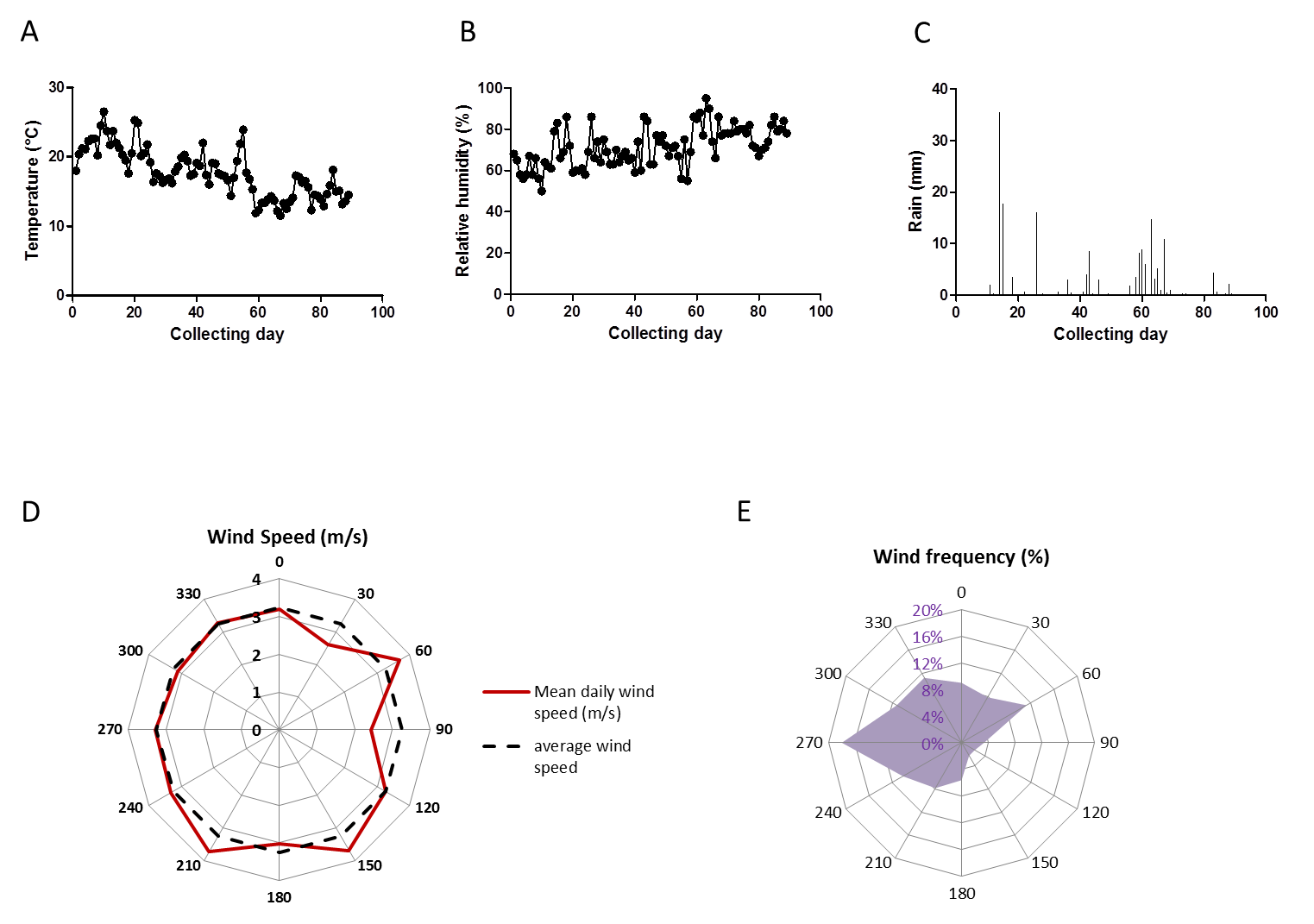


**Figure S2**. **Meteorological conditions during the sampling period.** **A** Temperature. **B** Relative humidity. **C** Rain. **D** Wind speed. **E** Wind direction and frequency.


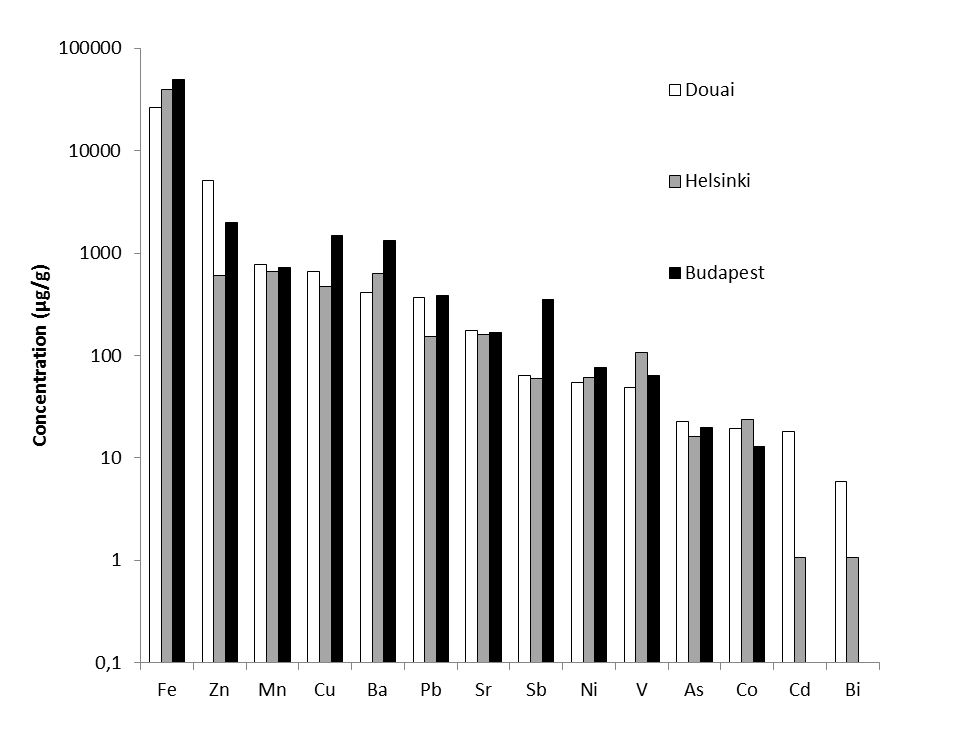


**Figure S3. Comparison of trace element concentrations in cPM^D^ collected at Douai and coarse PM collected in Helsinki, Finland [30] and Budapest, Hungary [31].**


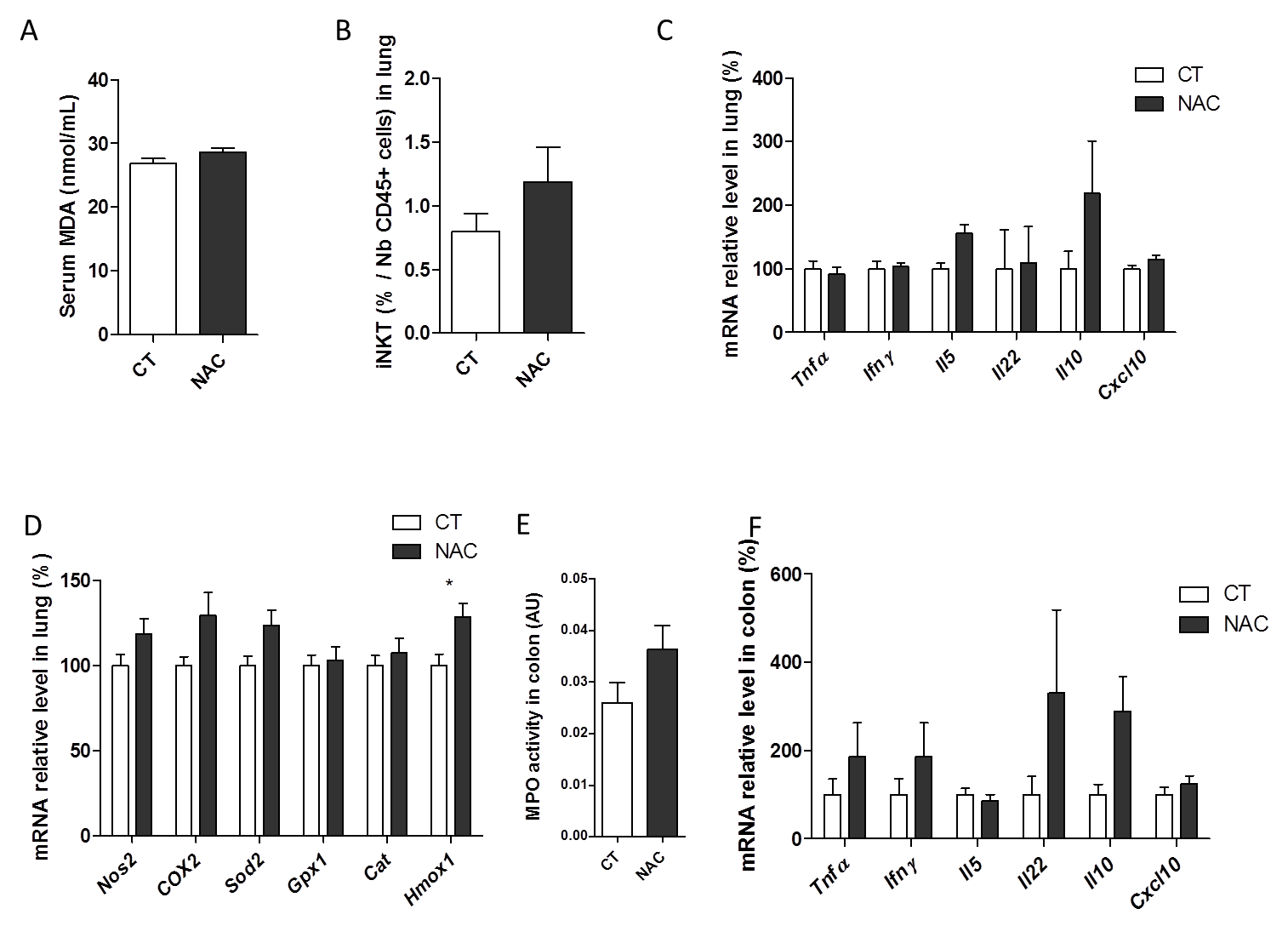


**Figure S4**. **Effects of NAC in the basal condition.** All mice inhaled sterile water. Mice that received NAC (15 µg/kg/day for 14 days) in drinking water were compared with CT mice that did not receive NAC in drinking water. **A** Serum MDA levels. **B** iNKT cell count measured by flow cytometry. **C** Quantitative PCR (qPCR) analysis of cytokine and chemokine mRNA levels in the lung. **D** qPCR analysis of mRNA levels of oxidative stress markers in the lung. **E** MPO activity in the colon. **F** qPCR analysis of cytokine and chemokine mRNA levels in the colon. Data are presented as mean ± SEM. *p<0.05, Mann-Whitney U test.
